# Supplementary material for: Genetic Factors Associated with COPD Depend on the Ancestral Caucasian/Amerindian Component in the Mexican Population
Source: Diagnostics (Basel). 2021 Mar 27;11(4):599. doi: 10.3390/diagnostics11040599 (PMC8067148; doi:10.3390/diagnostics11040599)
Supplement: Supplementary file 1 [file diagnostics-11-00599-s001.pdf]

Supplementary table S1. SNPs included in this study

Chr	GS	LN	MA	MAF
1	EPHX1	rs1051740	G	0.419
1	EPHX1	rs1051741	A	0.047
1	EPHX1	rs1877724	A	0.323
1	EPHX1	rs2234922	G	0.099
1	EPHX1	rs2260863	C	0.174
1	EPHX1	rs2671264	C	0.009
1	EPHX1	rs2671267	A	0.05
1	EPHX1	rs2671272	A	0.173
1	EPHX1	rs2854450	A	0.159
1	EPHX1	rs3738043	G	0.206
1	EPHX1	rs3753658	A	0.106
1	EPHX1	rs4149229	G	0
1	EPHX1	rs4653694	C	0.297
1	IL6R	rs10752641	C	0.211
1	IL6R	rs11265610	G	0.006
1	IL6R	rs12090237	A	0.011
1	IL6R	rs1386821	C	0.113
1	IL6R	rs28730733	C	0.005
1	IL6R	rs4072391	A	0.191
1	IL6R	rs4129267	A	0.498
1	IL6R	rs4845626	A	0.106
1	IL6R	rs6427641	A	0.425
1	IL6R	rs6684439	G	0.494
1	IL6R	rs7549250	G	0.393
2	GNLY	rs10188985	A	0.424
2	GNLY	rs10209258	A	0.015
2	GNLY	rs11887686	A	0.058
2	GNLY	rs12151621	A	0.148
2	GNLY	rs12989528	G	0.147
2	GNLY	rs17026540	A	0.437
2	GNLY	rs2043760	A	0.454
2	GNLY	rs4240201	A	0.378
2	GNLY	rs4525696	G	0.089
2	GNLY	rs4832183	G	0.479
2	GNLY	rs6547631	A	0.449
2	GNLY	rs6547632	A	0.454
2	GNLY	rs6732835	G	0.002
2	SERPINE2	rs10153617	A	0.005
2	SERPINE2	rs10164837	G	0.143
2	SERPINE2	rs10191694	A	0.217
2	SERPINE2	rs10194024	C	0.44
2	SERPINE2	rs11695803	A	0.17
2	SERPINE2	rs11884535	G	0.32
2	SERPINE2	rs12436	A	0.196
2	SERPINE2	rs12475117	G	0.034
2	SERPINE2	rs13008520	A	0.095
2	SERPINE2	rs13022548	C	0.092
2	SERPINE2	rs13393673	G	0.15
2	SERPINE2	rs1438830	A	0.035
2	SERPINE2	rs1438831	A	0.212
2	SERPINE2	rs16865390	A	0.274
2	SERPINE2	rs16865421	G	0.05
2	SERPINE2	rs16865497	A	0.002
2	SERPINE2	rs16865500	A	0.005
2	SERPINE2	rs16865507	A	0.002
2	SERPINE2	rs1866151	C	0.043
2	SERPINE2	rs1866153	A	0.115
6	TNF	rs1800629	A	0.063
6	TNF	rs2228088	A	0.005
6	TNF	rs361525	A	0.05
7	HIP1	rs10230391	C	0
7	HIP1	rs1167794	A	0.07
7	HIP1	rs1167795	G	0.48
7	HIP1	rs1167798	G	0.072
7	HIP1	rs1167801	G	0.072
7	HIP1	rs1167802	C	0.442
7	HIP1	rs12532457	A	0.008
7	HIP1	rs12535096	A	0.374
7	HIP1	rs12535903	A	0.078
7	HIP1	rs17149023	T	0
7	HIP1	rs6960211	G	0.005
7	HIP1	rs794355	A	0.272
7	HIP1	rs794356	A	0.451
7	IL6	rs13306435	A	0.133
7	IL6	rs1548216	C	0.086
7	IL6	rs1554606	A	0.252
7	IL6	rs1800796	G	0.292
7	IL6	rs2066992	A	0.291
7	IL6	rs2069830	A	0.008
7	IL6	rs2069832	A	0.162
7	IL6	rs2069838	A	0.003
7	IL6	rs2069840	C	0.333
7	IL6	rs2069842	A	0.011
7	IL6	rs2069849	A	0.083
7	IL6	rs2069852	A	0.275
7	SERPINE1	rs1050813	A	0.124
7	SERPINE1	rs2070682	G	0.301
7	SERPINE1	rs2227631	A	0.358
7	SERPINE1	rs2227636	A	0.003
7	SERPINE1	rs2227669	G	0
7	SERPINE1	rs2227672	A	0.078
7	SERPINE1	rs2227685	C	0
7	SERPINE1	rs2227693	G	0.005
7	SERPINE1	rs2227695	A	0
7	SERPINE1	rs2227714	A	0.02
7	SERPINE1	rs6090	A	0.072
7	SERPINE1	rs6950982	G	0.38
8	CHRNB3	rs13277254	G	0.242
8	CHRNB3	rs13280604	G	0.239
8	CHRNB3	rs6474412	G	0.24
8	CHRNB3	rs6474413	G	0.249
9	TNFSF8	rs1006026	A	0.494
9	TNFSF8	rs10759743	G	0.174
9	TNFSF8	rs10817689	G	0.338
9	TNFSF8	rs10817691	C	0.356
9	TNFSF8	rs10982467	A	0.347
9	TNFSF8	rs11791315	A	0.113
9	TNFSF8	rs12337739	T	0.265
9	TNFSF8	rs1322055	G	0.109
9	TNFSF8	rs1322062	A	0.008
9	TNFSF8	rs1555457	A	0.21
9	TNFSF8	rs17292115	C	0.236
9	TNFSF8	rs2181033	G	0.457
9	TNFSF8	rs3181350	G	0.054

2	*SERPINE2*	rs2083121	G	0.035
2	*SERPINE2*	rs2118409	C	0.305
2	*SERPINE2*	rs3795879	A	0.153
2	*SERPINE2*	rs3820766	A	0.031
2	*SERPINE2*	rs4368321	A	0.148
2	*SERPINE2*	rs4674839	A	0.225
2	*SERPINE2*	rs4674846	A	0.035
2	*SERPINE2*	rs6436459	A	0.261
2	*SERPINE2*	rs6708287	C	0.037
2	*SERPINE2*	rs6712954	A	0.291
2	*SERPINE2*	rs6734100	G	0.133
2	*SERPINE2*	rs6738983	A	0.471
2	*SERPINE2*	rs729631	C	0.153
2	*SERPINE2*	rs7560399	A	0.006
2	*SERPINE2*	rs7579646	A	0.126
2	*SERPINE2*	rs7597833	G	0.428
2	*SERPINE2*	rs7605945	G	0.413
2	*SERPINE2*	rs7608941	C	0.413
2	*SERPINE2*	rs861442	A	0.252
2	*SERPINE2*	rs920250	C	0
2	*SERPINE2*	rs975278	A	0.154
2	*SFTPB*	rs10204426	C	0.448
2	*SFTPB*	rs1030862	G	0.416
2	*SFTPB*	rs1130866	A	0.431
2	*SFTPB*	rs2040349	C	0.355
2	*SFTPB*	rs3024791	A	0.104
2	*SFTPB*	rs3024831	G	0.163
2	*SFTPB*	rs7316	G	0.196
2	*SFTPB*	rs934774	A	0.398
3	*PDZRN3*	rs1022170	C	0.488
3	*PDZRN3*	rs1039955	G	0.486
3	*PDZRN3*	rs1039956	A	0.48
3	*PDZRN3*	rs11918578	A	0.483
3	*PDZRN3*	rs12631241	C	0.116
3	*PDZRN3*	rs1355467	G	0.479
3	*PDZRN3*	rs1511520	G	0.329
3	*PDZRN3*	rs1511521	A	0.489
3	*PDZRN3*	rs1511531	A	0.489
3	*PDZRN3*	rs1543070	G	0.463
3	*PDZRN3*	rs1567573	C	0.459
3	*PDZRN3*	rs1877252	G	0.459
3	*PDZRN3*	rs2036893	A	0.491
3	*PDZRN3*	rs2137064	A	0.494
3	*PDZRN3*	rs4677326	C	0.483
3	*PDZRN3*	rs4677328	A	0.463
3	*PDZRN3*	rs6777769	C	0.46
3	*PDZRN3*	rs6781766	A	0.324
3	*PDZRN3*	rs6787034	G	0.486
3	*PDZRN3*	rs7645388	A	0.488
3	*PDZRN3*	rs925431	A	0.489
3	*PDZRN3*	rs9310276	A	0.33
3	*PDZRN3*	rs9310278	A	0.327
3	*PDZRN3*	rs9310279	G	0.494
3	*PDZRN3*	rs932017	G	0.414
3	*PDZRN3*	rs952708	A	0.498
3	*PDZRN3*	rs970543	G	0.278
3	*PDZRN3*	rs9790092	A	0.462
3	*PDZRN3*	rs9847694	A	0.486
3	*PDZRN3*	rs994960	A	0.457
4	*GYPA*	rs1132787	A	0.217
4	*GYPA*	rs12499911	G	0.02

9	*TNFSF8*	rs3181354	G	0.254
9	*TNFSF8*	rs3181358	A	0.012
9	*TNFSF8*	rs3181360	A	0.278
9	*TNFSF8*	rs3789878	C	0.234
9	*TNFSF8*	rs3789879	A	0.268
9	*TNFSF8*	rs6478121	G	0.337
9	*TNFSF8*	rs7026285	G	0.002
9	*TNFSF8*	rs7854103	G	0.041
9	*TNFSF8*	rs881172	A	0.339
9	*TNFSF8*	rs927373	A	0.457
9	*TNFSF8*	rs979584	C	0.134
10	*SFTPD*	rs17878336	C	0.015
10	*SFTPD*	rs17878740	A	0.002
10	*SFTPD*	rs1885551	G	0.268
10	*SFTPD*	rs1885553	G	0.182
10	*SFTPD*	rs1923539	A	0.144
10	*SFTPD*	rs2146192	G	0.268
10	*SFTPD*	rs2181204	G	0.274
10	*SFTPD*	rs2245121	A	0.301
10	*SFTPD*	rs2819096	A	0.43
10	*SFTPD*	rs4469829	A	0.002
10	*SFTPD*	rs6413520	G	0.024
10	*SFTPD*	rs6413523	A	0.046
10	*SFTPD*	rs7078012	A	0.121
10	*SFTPD*	rs726014	G	0.274
10	*SFTPD*	rs911887	G	0.26
13	*FOXO1*	rs12583418	A	0.477
13	*FOXO1*	rs2721066	G	0.479
13	*FOXO1*	rs3892360	C	0.474
13	*FOXO1*	rs4325427	A	0.463
13	*FOXO1*	rs4429172	A	0.472
13	*FOXO1*	rs4603422	G	0.483
13	*FOXO1*	rs4943795	G	0.471
13	*FOXO1*	rs7330614	A	0.477
13	*FOXO1*	rs7335520	A	0.479
13	*FOXO1*	rs7338669	G	0.48
13	*FOXO1*	rs7986407	G	0.471
13	*FOXO1*	rs9549236	G	0.479
13	*FOXO1*	rs9566553	G	0.48
15	*CHRNA3*	rs1051730	A	0.234
15	*CHRNA3*	rs11637630	A	0.434
15	*CHRNA3*	rs3743078	C	0.415
15	*CHRNA3*	rs578776	G	0.407
15	*CHRNA3*	rs6495308	A	0.431
15	*CHRNA5*	rs16969968	A	0.216
15	*CHRNA5*	rs17486278	C	0.24
15	*CHRNA5*	rs2036527	A	0.223
15	*CHRNA5*	rs569207	G	0.437
15	*CHRNA5*	rs588765	A	0.2
15	*CHRNA5*	rs637137	A	0.436
15	*CHRNA5*	rs684513	C	0.414
15	*CHRNB4*	rs11636605	G	0.434
15	*CHRNB4*	rs12440014	C	0.457
15	*CHRNB4*	rs1316971	A	0.343
15	*CHRNB4*	rs17487223	A	0.243
16	*MMP15*	rs1050779	C	0.408
16	*MMP15*	rs11076213	A	0.497
16	*MMP15*	rs12924451	A	0.373
16	*MMP15*	rs12929002	A	0.408
16	*MMP15*	rs1436426	G	0.417
16	*MMP15*	rs2118018	G	0.426

4	*GYPA*	rs13103731	A	0.249
4	*GYPA*	rs13105210	A	0.291
4	*GYPA*	rs13125760	G	0.433
4	*GYPA*	rs1505771	A	0.219
4	*GYPA*	rs2719341	A	0.272
4	*GYPA*	rs4031150	A	0.193
4	*GYPA*	rs4130880	G	0.084
4	*GYPA*	rs4256191	C	0.223
4	*GYPA*	rs4321584	G	0.221
4	*GYPA*	rs4374581	A	0.067
4	*GYPA*	rs4469024	G	0.153
4	*GYPA*	rs6537278	A	0.193
4	*GYPA*	rs6828489	A	0.219
4	*GYPA*	rs6844670	A	0.339
4	*GYPA*	rs6857262	C	0.295
4	*GYPA*	rs6857303	A	0.284
4	*GYPA*	rs6858668	G	0.014
4	*GYPA*	rs7377575	A	0.217
4	*GYPA*	rs7655235	A	0.222
4	*HHIP*	rs1489758	A	0.209
4	*HHIP*	rs1812175	A	0.185
4	*HHIP*	rs2307057	G	0.035
4	*HHIP*	rs2575580	G	0.113
4	*HHIP*	rs6812389	G	0.48
4	*HHIP*	rs6850355	A	0
4	*HHIP*	rs6857302	A	0.313
4	*HHIP*	rs7689420	A	0.185
4	*HHIP*	rs982902	A	0.482
5	*ADAM19*	rs10067096	C	0.113
5	*ADAM19*	rs10078120	A	0.011
5	*ADAM19*	rs10404	A	0.211
5	*ADAM19*	rs11134778	A	0.096
5	*ADAM19*	rs11134799	G	0.048
5	*ADAM19*	rs11134804	C	0.002
5	*ADAM19*	rs11134819	A	0.08
5	*ADAM19*	rs11465283	A	0.102
5	*ADAM19*	rs11466776	A	0.096
5	*ADAM19*	rs11466782	G	0.269
5	*ADAM19*	rs11466793	A	0.006
5	*ADAM19*	rs11466812	C	0
5	*ADAM19*	rs11466819	A	0.003
5	*ADAM19*	rs11466824	G	0
5	*ADAM19*	rs11466826	G	0.002
5	*ADAM19*	rs11748149	A	0.034
5	*ADAM19*	rs12518544	G	0.005
5	*ADAM19*	rs13179607	C	0.258
5	*ADAM19*	rs13353878	A	0.121
5	*ADAM19*	rs1422794	C	0.315
5	*ADAM19*	rs1422795	G	0.474
5	*ADAM19*	rs17054692	G	0.098
5	*ADAM19*	rs17054697	A	0.113
5	*ADAM19*	rs17054709	A	0.139
5	*ADAM19*	rs17601035	G	0.049
5	*ADAM19*	rs17659250	A	0.208
5	*ADAM19*	rs1990950	A	0.275
5	*ADAM19*	rs2042247	A	0.442
5	*ADAM19*	rs2277027	C	0.477
5	*ADAM19*	rs2287749	A	0.06
5	*ADAM19*	rs3734032	A	0.135
5	*ADAM19*	rs3822585	G	0.323
5	*ADAM19*	rs4331881	A	0.474
16	*MMP15*	rs2241773	A	0.408
16	*MMP15*	rs2304488	G	0.434
16	*MMP15*	rs3784905	G	0.011
16	*MMP15*	rs3784909	A	0.032
16	*MMP15*	rs4784887	G	0.451
16	*MMP15*	rs8058147	G	0.012
16	*MMP15*	rs8064087	A	0.006
16	*MMP15*	rs9924342	A	0
16	*MMP15*	rs9926693	G	0.443
19	*CYP2A6*	rs10418304	G	0.002
19	*CYP2A6*	rs11670760	G	0.44
19	*CYP2A6*	rs11879413	A	0.156
19	*CYP2A6*	rs2644890	A	0.067
19	*CYP2A6*	rs28399456	G	0
19	*CYP2A6*	rs4105144	A	0.379
19	*CYP2A6*	rs7251418	A	0.179
19	*CYP2A6*	rs8192729	A	0.028
19	*CYP2B6*	rs1042389	G	0.129
19	*CYP2B6*	rs10426235	A	0.141
19	*CYP2B6*	rs11672911		
19	*CYP2B6*	rs11673270	C	0.323
19	*CYP2B6*	rs12721649	A	0.008
19	*CYP2B6*	rs1552223	G	0.182
19	*CYP2B6*	rs16974796	C	0.005
19	*CYP2B6*	rs1808682	A	0.235
19	*CYP2B6*	rs1872125	G	0.316
19	*CYP2B6*	rs2014141	A	0.196
19	*CYP2B6*	rs2279344	G	0.182
19	*CYP2B6*	rs2279345	A	0.188
19	*CYP2B6*	rs2291287	G	0.022
19	*CYP2B6*	rs28399499	G	0.006
19	*CYP2B6*	rs28399502	C	0
19	*CYP2B6*	rs36060847	G	0
19	*CYP2B6*	rs3760657	G	0.235
19	*CYP2B6*	rs4803415	A	0.235
19	*CYP2B6*	rs4803416	G	0.023
19	*CYP2B6*	rs4803417	C	0.206
19	*CYP2B6*	rs6508964	G	0.195
19	*CYP2B6*	rs707265	A	0.192
19	*CYP2B6*	rs7250601	C	0.335
19	*CYP2B6*	rs7260329	A	0.472
19	*CYP2B6*	rs8100458	G	0.468
19	*CYP2B6*	rs8113196	A	0.194
19	*CYP2B6*	rs8192711	A	0.025
19	*CYP2B6*	rs8192712	G	0.232
19	*EGLN2*	rs11083567	A	0.202
19	*EGLN2*	rs11083568	A	0.198
19	EGLN2	rs11879672	A	0.057
19	*EGLN2*	rs16974537	A	0.003
19	*EGLN2*	rs2545771	A	0.021
19	*EGLN2*	rs2604866	C	0.008
19	*EGLN2*	rs3733829	A	0.485
19	*EGLN2*	rs4803369	G	0.485
19	*EGLN2*	rs8111961	A	0.002
19	*GPATCH1*	rs10416265	G	0.376
19	*GPATCH1*	rs10420258	A	0.126
19	*GPATCH1*	rs13346412	G	0.066
19	*GPATCH1*	rs16967824	A	0.006
19	*GPATCH1*	rs1981818	A	0.33
19	*GPATCH1*	rs2287679	G	0.292
19	*GPATCH1*	rs2287681	C	0.492

5	*ADAM19*	rs4461616	C	0.2
5	*ADAM19*	rs6861910	A	0.005
5	*ADAM19*	rs6866363	G	0.457
5	*ADAM19*	rs6875485	C	0.11
5	*ADAM19*	rs6895849	A	0.05
5	*ADAM19*	rs6896064	C	0.304
5	*ADAM19*	rs7707786	C	0.116
5	*ADAM19*	rs7724199	A	0.055
5	*ADAM19*	rs7725069	A	0.166
5	*ADAM19*	rs7725846	A	0.323
5	*ADAM19*	rs9313633	T	0.11
5	*ADAM19*	rs9313634	G	0.451
5	*ADRB2*	rs1042714	C	0.198
5	*ADRB2*	rs1042717	A	0.357
5	*ADRB2*	rs11168066	C	0.226
5	*ADRB2*	rs11959615	A	0.225
5	*ADRB2*	rs2082382	G	0.193
5	*ADRB2*	rs3857420	A	0.383
6	*GPR126*	rs1040526	G	0.306
6	*GPR126*	rs11155242	C	0.179
6	*GPR126*	rs12664563	G	0.193
6	*GPR126*	rs1329705	A	0.174
6	*GPR126*	rs1360194	A	0.305
6	*GPR126*	rs155259	A	0.31
6	*GPR126*	rs1928528	C	0.194
6	*GPR126*	rs2294764	A	0.301
6	*GPR126*	rs2294775	C	0.195
6	*GPR126*	rs262114	A	0.311
6	*GPR126*	rs262115	G	0.463
6	*GPR126*	rs262119	G	0.314
6	*GPR126*	rs262129	G	0.313
6	*GPR126*	rs263178	A	0.293
6	*GPR126*	rs263179	G	0.306
6	*GPR126*	rs3748069	G	0.31
6	*GPR126*	rs3817928	G	0.179
6	*GPR126*	rs4896582	A	0.454
6	*GPR126*	rs611802	C	0.315
6	*GPR126*	rs6570509	A	0.307
6	*GPR126*	rs6570511	A	0.199
6	*GPR126*	rs6906468	G	0.193
6	*GPR126*	rs6937121	C	0.314
6	*GPR126*	rs7753012	C	0.455
6	*GPR126*	rs7755109	G	0.306
6	*GPR126*	rs7763064	A	0.308
6	*GPR126*	rs7774095	A	0.289
6	*GPR126*	rs7776356	G	0.194
6	*GPR126*	rs962554	G	0.304
19	*GPATCH1*	rs3786931	A	0.07
19	*GPATCH1*	rs3786934	G	0.159
19	*GPATCH1*	rs3826906	A	0.149
19	*TGFB1*	rs11466324	A	0.002
19	*TGFB1*	rs11466338	G	0.028
19	*TGFB1*	rs2014015	C	0
19	*TGFB1*	rs2241715	A	0.442
19	*TGFB1*	rs2241716	A	0.018
19	*TGFB1*	rs8179181	A	0.125
20	*ADAM33*	rs2853210	A	0.206
20	*ADAM33*	rs3746631	G	0.096
20	*ADAM33*	rs3918392	G	0.029
20	*ADAM33*	rs3918395	A	0.072
20	*ADAM33*	rs487377	A	0.275
20	*ADAM33*	rs511898	A	0.336
20	*ADAM33*	rs512625	A	0.219
20	*ADAM33*	rs528557	C	0.171
20	*ADAM33*	rs543749	A	0.133
20	*ADAM33*	rs554743	G	0.164
20	*ADAM33*	rs598418	G	0.407
20	*ADAM33*	rs612709	A	0.087
21	*ADARB1*	rs1051385	G	0.046
21	*ADARB1*	rs12329821	A	0.021
21	*ADARB1*	rs1556318	A	0.179
21	*ADARB1*	rs17004752	G	0.023
21	*ADARB1*	rs17004755	T	0.017
21	*ADARB1*	rs2150439	G	0.394
21	*ADARB1*	rs2225434	G	0.428
21	*ADARB1*	rs2838806	A	0.422
21	*ADARB1*	rs2838807	A	0.396
21	*ADARB1*	rs2838809	A	0.173
21	*ADARB1*	rs2838813	A	0.422
21	*ADARB1*	rs2838815	A	0.425
21	*ADARB1*	rs2838816	A	0.175
21	*ADARB1*	rs2838818	G	0.463
21	*ADARB1*	rs2838820	A	0.445
21	*ADARB1*	rs2838821	A	0.439
21	*ADARB1*	rs2838824	A	0.119
21	*ADARB1*	rs3753016	A	0.404
21	*ADARB1*	rs4592938	A	0.015
21	*ADARB1*	rs4819035	C	0.144
21	*ADARB1*	rs6518216	C	0.192
21	*ADARB1*	rs6518219	T	0.121
21	*ADARB1*	rs748559	A	0.02
21	*ADARB1*	rs914210	G	0.361
21	*ADARB1*	rs9980546	A	0.031
21	*ADARB1*	rs9981665	A	0.021

Chr; chromosome. GS; Gene symbol. LN; Locus name. MA; minor allele in the study population. MAF; minor allele frequency.
